# Cryptococcal Meningitis Diagnosed With Lumbar Puncture in an Emergency Department Patient With Acute Delirium

**DOI:** 10.7759/cureus.45603

**Published:** 2023-09-20

**Authors:** Ambra Palushi, Bryan Choi

**Affiliations:** 1 Emergency Medicine, American University of Antigua, Coolidge, ATG; 2 Emergency Medicine, Philadelphia College of Osteopathic Medicine, Dover, USA

**Keywords:** infectious disease, delirium, lumbar puncture, emergency department, cryptococcal meningitis

## Abstract

Cryptococcal meningitis is a life-threatening fungal infection of the central nervous system. It primarily affects immunocompromised individuals, particularly those with advanced HIV/AIDS, but can occur in people of all ages. Risk factors include solid organ transplantation, hematological malignancies, immunosuppressive medications, and primary immunodeficiency disorders. We describe a case of a 68-year-old male with atypical risk factors for cryptococcal meningitis who presented acutely delirious with a negative diagnostic workup aside from a positive cerebrospinal fluid (CSF) meningitis screen reporting *Cryptococcus neoformans/gattii. *While the rate of lumbar puncture (LP) in the ED has drastically decreased over the years, we emphasize the importance of considering its diagnostic value in emergency department settings in patients with atypical presentations.

## Introduction

The overall prevalence of cryptococcal meningitis in the United States is fairly low, with the greatest incidence being in regions with a high prevalence of HIV/AIDS and in resource-limited settings [1]. The most common presenting signs and symptoms include fever, headache, altered mental status, and neck stiffness [2]. Infection typically starts with the inhalation of *Cryptococcus neoformans* or *Cryptococcus gattii* spores, which disseminate to the central nervous system (CNS), leading to meningitis [3]. Left untreated, infection can lead to coma, seizures, and death, with a mortality rate of 10%-30% [4,5]. Laboratory diagnosis is typically achieved via cryptococcal antigen testing or through newer methods such as multiplex polymerase chain reaction (PCR) assays, however, still relies on the successful performance of a lumbar puncture (LP) to obtain the necessary cerebrospinal fluid (CSF) [6]. In the emergency department, the utilization of LP has drastically changed over the years but remains a key workup component in the setting of patients who present with altered mental status or delirium with an unknown underlying cause based on other negative diagnostic findings.

## Case presentation

A 68-year-old man was brought to our community teaching hospital Emergency Department (ED) via ambulance after his family noticed an acute, significant change in his behavior and cognition, starting the night before. His medical history included hypertension, hepatitis C not on active treatment, type 2 diabetes, and chronic neck and back pain with a history of a cervical fusion in 2019. Severely delirious, the patient was unable to follow any commands or answer any questions; verbalization was limited to gibberish alternating with anguished moans, and he continuously thrashed around while flailing his limbs. Intramuscular lorazepam (2mg once) and droperidol (2.5mg once) administration slowed his movements but did not improve his cognition. His physical exam showed the following vitals: blood pressure: 151/103, heart rate: 133, respiratory rate: 22, oral temperature: 38.6º C (98.2 F), oxygen saturation: 98% on room air. In general, he appeared to be in pain, diaphoretic, and agitated. There were no signs of obvious external injury. His ability to participate in a neurologic exam was substantially limited, but we were able to determine normal cranial nerve function with the exception of olfaction, normal and symmetric motor strength both proximally and distally for all extremities, and 2+ patellar reflexes. He was unable to answer any questions, and we were unable to assess for sensory deficits, visual field cuts, or orientation. We performed a comprehensive metabolic and infectious workup, which demonstrated a relatively mild lactic acidosis (3.0 mmol/L), a mild leukocytosis (WBC 11.9 K/μL), elevated CK (1,802 U/L), and no evidence of overt pneumonia on Chest x-ray or urinary tract infection. Computed along with additional tests, we obtained informed consent from the patient’s family to perform an LP to rule out meningitis. After two attempts, we obtained clear CSF, which on initial cell count seemed to rule out overt bacterial meningitis with a cell count showing only 468 RBC and 2 WBC. Protein and Glucose were elevated at 65 mg/dL and 95 mg/dL, respectively. An opening pressure could not be obtained due to the patient’s agitation. We started the patient on Vancomycin (1,000 mg IV every 12 hours), Ampicillin (2g IV every six hours), and Ceftriaxone (2g IV every 12 hours) and admitted him to our hospitalist service. Shortly before he left the ED, his CSF meningitis/encephalitis panel (BioFire FilmArray, BioFire Diagnostics, Salt Lake City, UT), resulted positive for *C. neoformans*/*gattii*.

The patient spent 17 days in the hospital, during which he was evaluated by multiple consulting services including Neurology, Nephrology, and Infectious Disease. He tested negative for HIV. Serum cryptococcal antigen testing was negative, as were his blood cultures. He underwent a 14-day course of liposomal amphotericin B (Ambisome, 5 mg/kg IV every 24 hours) and flucytosine (25mg/kg by mouth every 6 hours); Vancomycin and Ampicillin were discontinued upon discovery of the CSF results; however, Ceftriaxone was continued. He was also started on a seven-day course of Topiramate (50mg by mouth twice daily) for seizure prophylaxis. The patient’s complications included acute tubular necrosis and acute kidney injury, which were attributed to having received Amphotericin B and Topiramate. By the time of his discharge, he was alert, oriented, and ambulatory. He was prescribed an eight-week course of Fluconazole (400mg by mouth once daily), with follow-up appointments planned with Nephrology, Infectious Disease, and Primary Care.

## Discussion

In a clinical setting, it is vital to obtain prompt diagnosis for rare but dangerous CNS infections like cryptococcal meningitis. Timely diagnosis allows for the initiation of appropriate antifungal therapy as well as for the involvement of consult services and management of complications [7]. Broad-spectrum antibiotics administered in the ED would not have successfully addressed the underlying cause of this patient’s delirium. Despite being the gold standard for diagnosis of meningitis [8], LPs have become less common in ED settings, as evidenced by a decreasing share of procedures (from 21.8% to 15.2%) performed by emergency physicians from 2010 to 2018, with increasing share to radiologists (from 45.7% to 52.3%) [9]. LPs are time-consuming procedures [10], and ED overcrowding has placed an ever-increasing demand on clinicians’ time [11]. However, as this case demonstrates, an ED LP has value especially when the diagnostic workup for severe delirium has not yielded a ready explanation. A fungal CNS infection was initially low on the list of likely differential diagnoses because this patient did not have HIV, had no history suggesting fungal spore exposure, and was not on any immunocompromising medications. The typical presentation of cryptococcal meningitis is also smoldering in nature and worsens over weeks, as opposed to an abrupt change in mentation [1]. Type 2 diabetes, however, has been shown to be a potential risk factor for cryptococcal infection due to causing immune dysfunction [12], and the same is true for liver cirrhosis [13]. It is also possible that this patient’s change in mentation could have happened more insidiously, with his family only noticing acutely due to his agitation. With this patient’s risk factors and many of the more likely causes of delirium ruled out through his workup, an LP was the next logical step for achieving a timely diagnosis, even though this patient would have likely gotten an LP as an inpatient, regardless.

## Conclusions

Acute delirium has a wide differential diagnosis, and CNS infections causing meningitis and encephalitis are common causes of acute changes in mental status. Fungal meningitis is an atypical cause of CNS infection and usually affects immunocompromised patients, but can also affect those with normal immune systems, even though the probability is low. An LP obtained from the ED can be the quickest way to obtain critical diagnostic information, even if life-threatening etiologies like bacterial meningitis are considered to be unlikely. When a diagnostic workup for typical items on the differential diagnosis fails to identify a compelling reason for delirium in an ED setting, an LP should be considered.
